# Micronutrients in Pregnancy in Low- and Middle-Income Countries

**DOI:** 10.3390/nu7031744

**Published:** 2015-03-10

**Authors:** Ian Darnton-Hill, Uzonna C. Mkparu

**Affiliations:** 1 The Boden Institute of Obesity, Nutrition, Exercise & Eating Disorders, University of Sydney, NSW 2006, Australia; 2 The Friedman School of Nutrition Science and Policy, Tufts University, Medford, MA 021111, USA; 3 Columbia University Medical Center, Institute of Human Nutrition, New York, NY 10027, USA; E-Mail: ucm2103@columbia.edu

**Keywords:** adolescents, pregnant women, pregnancy, micronutrients, antenatal care, low- and middle-income countries (LMIC)

## Abstract

Pregnancy is one of the more important periods in life when increased micronutrients, and macronutrients are most needed by the body; both for the health and well-being of the mother and for the growing foetus and newborn child. This brief review aims to identify the micronutrients (vitamins and minerals) likely to be deficient in women of reproductive age in Low- and Middle-Income Countries (LMIC), especially during pregnancy, and the impact of such deficiencies. A global prevalence of some two billion people at risk of micronutrient deficiencies, and multiple micronutrient deficiencies of many pregnant women in LMIC underline the urgency to establishing the optimal recommendations, including for delivery. It has long been recognized that adequate iron is important for best reproductive outcomes, including gestational cognitive development. Similarly, iodine and calcium have been recognized for their roles in development of the foetus/neonate. Less clear effects of deficiencies of zinc, copper, magnesium and selenium have been reported. Folate sufficiency periconceptionally is recognized both by the practice of providing folic acid in antenatal iron/folic acid supplementation and by increasing numbers of countries fortifying flours with folic acid. Other vitamins likely to be important include vitamins B12, D and A with the water-soluble vitamins generally less likely to be a problem. Epigenetic influences and the likely influence of micronutrient deficiencies on foetal origins of adult chronic diseases are currently being clarified. Micronutrients may have other more subtle, unrecognized effects. The necessity for improved diets and health and sanitation are consistently recommended, although these are not always available to many of the world’s pregnant women. Consequently, supplementation programmes, fortification of staples and condiments, and nutrition and health support need to be scaled-up, supported by social and cultural measures. Because of the life-long influences on reproductive outcomes, including inter-generational ones, both clinical and public health measures need to ensure adequate micronutrient intakes during pregnancy, but also during adolescence, the first few years of life, and during lactation. Many antenatal programmes are not currently achieving this. We aim to address the need for micronutrients during pregnancy, the importance of micronutrient deficiencies during gestation and before, and propose the scaling-up of clinical and public health approaches that achieve healthier pregnancies and improved pregnancy outcomes.

## 1. Introduction

Optimal outcomes of pregnancy and their importance to the mother, the future child, families and societies, is contingent on appropriate care, adequate antenatal preparation and sufficient nutrition. The consequences of antenatal nutritional deficiencies can be devastating to the mother, child and effect future generations. As such, it is critical that expectant mothers enter pregnancy with the best possible macronutrient and micronutrient status and then receive adequate antenatal nutrition for their health, and for the well-being of their offspring. This short review examines micronutrient deficiencies in women in Low and Middle Income Countries, and programmatic responses.

Maternal nutrition has profound effects on foetal growth, development, and subsequent infant birthweight, and the health and well-being of the woman herself [1]. Maternal undernutrition, maternal mortality rates, infant mortality and morbidity rates have declined since the 1990s as a result of increasing attention to improving the quality of the antenatal period and improving obstetric care and social change. However, there is still a great need for further improvements. The nutritional status and size of the pregnant woman is the result of past health and nutrition, including her own birth size and subsequent health and societal influences.

Poor dietary patterns, and options, in the periconceptional period are known to lead to pre-term delivery, shorter birth-length and earlier gestation [1] and poor potential neurodevelopmental outcomes for the foetus [2]. Given the impact of poor maternal diet, both public health and clinical measures need to be in place, especially in low socio-economic environments. These need to address all stages of the women’s life-cycle, and especially during the pregnancy. There is increased risk if that pregnancy occurs during adolescence, is spaced too closely to a preceding pregnancy, or is one of multiple pregnancies. Nutritional, dietary and health interventions need to be complemented by improved obstetric care and support, and exposure to “nutrition-sensitive” interventions such as access to education, improvement in women’s status and improved agricultural and environmental determinants [3].

While the global burden of diseases caused by deficiencies of micronutrients during pregnancy is relatively modest globally, the cumulative individual impact can be considerable. This is especially so for adolescent pregnancies and women of lower economic or minority status in low and middle-income economic settings [4]. The aim of this short review is to describe micronutrient deficiencies and programmes in LMIC where the vast majority of micronutrient deficiencies occur, and appropriate public health and maternal antenatal care in such settings to address such deficiencies. Where relevant, research literature from more affluent countries is also used.

## 2. Micronutrient Deficiencies in Women of Reproductive Age

Globally, approximately two billion people, the majority women and young children, are affected, by micronutrient deficiencies, with even higher rates during pregnancy [5]. Concurrent deficiencies of more than one or two micronutrients are well documented among young pregnant women, (and young children), especially in LMIC [6,7,8,9]. Deficiencies in maternal micronutrient status are a result of: poor quality diets; high fertility rates; repeated pregnancies; short inter-pregnancy intervals; and, increased physiological needs. These factors are aggravated by often inadequate health systems with poor capacity, by poverty and inequities, and by socio-cultural factors such as early marriage and adolescent pregnancies, and some traditional dietary practices [3,4,10,11,12]. A systematic review identifying all studies that had been published between 1988 and 2008 reporting on micronutrient intakes in women living in such environments, showed that for women, the reported mean/median intakes in over 50% of the studies were below the Estimated Average Requirements (EAR) for micronutrient intakes, except for vitamin A, vitamin C, and niacin, where the reported intakes were around a third of the EAR, 29%, 34% and 34% respectively [11].

Pregnancy during adolescence is a relatively common event in much of the world [13] and the young women are usually incomplete in their own growth and often deficient in micronutrients [14,15]. Pregnancies at this time will make reproductive outcomes more likely to be negative as well as impacting on the health, nutrition and well-being of the adolescent. Studies of micronutrient status in adolescents, including when pregnant, have found poor micronutrient intakes and status [14], including in the UK [16], and increased risk of small-for-gestational-age (SGA) and low birthweight [LBW] infants at birth [10]. Adolescent pregnancy, besides negatively affecting the young mother’s own growth and nutritional status [17], is associated with a 50% increased risk of stillbirths and neonatal deaths, and increased risk of preterm birth, low birthweight, and asphyxia [18]. A review assessing the association between inter-pregnancy intervals with maternal, newborn, and child health outcomes found that short inter-pregnancy intervals (<6 months) were also associated with a higher probability of maternal anaemia (32%) and stillbirths (40%) whereas longer intervals (>60 months) were associated with an increased risk of pre-eclampsia [19].

## 3. Micronutrients during Pregnancy

Information concerning vitamin and mineral metabolism and requirements during pregnancy are surprisingly imprecise, largely because of the complexity of maternal metabolism during pregnancy [4,20] and interactions between micronutrients. Overall nutrient requirements are increased during pregnancy due to the greater needs of the mother’s own increase in body tissue reserves and metabolic demands, and the development of placenta and the foetus [21,22]. Requirements for many, but not all, micronutrients also increase during pregnancy [21]. However the increased requirements will depend on existing nutritional status, rate of weight gain and availability of adequate nutrition and co-existing disease. For micronutrients especially, adequacy can be difficult to assess due to plasma volume increases and often-poor biomarkers [23,24]. Nutrient-binding proteins that transport micronutrients also demonstrate decreased concentrations [21].

*Iron and iron deficiency anaemia*: iron deficiency anaemia (IDA) leading to a decrease in oxygen carrying capacity is one of the most common pregnancy-related complications [23,25]. The majority of the 1.62 billion people currently affected by anaemia are women or young children [25]. The global prevalence in pregnant women has fallen only slightly since 1995 from 43% to 38% [25]. The global prevalence of severe anaemia on the other hand, which poses the greatest risk for maternal mortality, has shown a greater relative reduction but still only from 2.0% to 0.9%, and overall the risk is far higher in women of LMIC [25]. About half of all anaemia is estimated to be attributable to iron deficiency, depending on the geographic and disease environment. Much of the other (approximately) half is caused by diseases such as malaria, HIV and parasites, and by deficiencies of other micronutrients such as vitamin A, folate and zinc [22], again according to the local environments.

The apparent increased risk of anaemia during pregnancy is confounded by the plasma volume expansion at about six weeks into pregnancy [23], although red blood cell mass does not increase proportionately to the expanding plasma volume. Plasma volume increases by about 48% while red cell mass only increases by about 18% [23]. Iron deficiency itself, even before manifest as anaemia, affects both mother and child [23] and in the mother includes cognitive impairment, decreased physical activity and reduced immunity, and possibly more subtle impairments. Where there is real iron deficiency, this decreases the mother’s ability to synthesize hemoglobin and transport oxygen [26]. The foetus developing *in-utero* has no direct contact with the atmosphere and depends on the mother for oxygen, although foetal hemoglobin does also have a higher affinity for oxygen which helps to ensure that the developing foetus’ oxygen requirements can be met [27].

Young children who are the offspring of anaemic mothers, or are anaemic themselves, usually have poor development. A recent overview reported on a relatively recent meta-analysis that established the strong causal link between maternal iron deficiency and adverse outcomes [28]. Amongst other things, iron deficiency is thought to affect the optimal development of the foetal brain [20] and in mice at least, gestational iron deficiency of the mother differentially alters the structure and function of white and grey matter brain regions of the offspring [29]. A recent study found that psychosocial stimulation benefitted development in non-anaemic children but not in anaemic, iron-deficient children [30]. This would suggest in addition to iron treatment, children with IDA may require more intense or longer interventions than for young children neither anaemic nor iron deficient. There have been studies now with many years of follow-up that have demonstrated direct positive association between maternal Hb levels during pregnancy and educational achievements of off-spring later in life. One, from Finland, has demonstrated improvements 31 years later [31]. The study authors suggest that that iron prophylaxis even at fairly late stages of pregnancy may be beneficial for the offspring [31]. However, while iron deficiency is to be avoided in pregnancy, iron supplements and increased iron stores in the third trimester have been linked to maternal complications such as gestational diabetes and increased oxidative stress and risk of preeclampsia [32]. The author notes that anaemia and iron deficiency anaemia are not synonymous, including among low income and minority women in their reproductive years [32].

*Iodine*: Unlike most essential dietary nutrients, iodine status is not linked so much to socio-economic development but more to geography [33]. Its critical significance during pregnancy is, rather than on maternal health directly, due to the devastating impact on the foetus of deficiency, including cretinism and impaired growth. Nevertheless, reproductive outcomes are affected with increased risk of stillbirths, abortions, and congenital abnormalities. Maternal urinary iodine has also been positively associated with birth weight, length and head circumference in male offspring in a recent study of a Bangladeshi population of pregnant women [2], as well as the well-recognized impact on offspring cognitive impairment as described below.

*Calcium*: Calcium supplementation is associated with a reduction in pre-eclampsia as well as LBW and pre-term birth [18]. Gestational hypertensive disorders are the second main causes of maternal morbidity and mortality, as well as being associated with an increased risk of pre-term birth and foetal growth restriction [34]. As calcium supplementation during pregnancy reduces the incidence of gestational hypertension by 35%, pre-eclampsia by 52%–55% and pre-term births by 24% [35], the World Health Organization (WHO) now recommends 1.5 g to 2.0 g of elemental calcium per day for pregnant women with low dietary calcium intakes.

*Other minerals*: Other trace element deficiencies that have been described as possibly associated with complications in pregnancy, childbirth or foetal development include copper, magnesium, selenium and zinc [22]. While a Cochrane review found that zinc supplementation in pregnancy may result in a 14% reduction in preterm birth [36], this decrease was not accompanied by a similar reduction in stillbirths, neonatal death, SGA, or low birthweight. The Lancet Series on Maternal and Young Child Undernutrition concluded there is insufficient evidence at this point for policy to be made on zinc supplementation during pregnancy [18].

*Folate*: folate deficiency leading to megaloblastic anemia is the second most common cause of anaemia during pregnancy [23]. Folate, a B-vitamin, has an important role in the synthesis and maintenance of DNA and therefore has an increased requirement throughout pregnancy supporting optimal growth and development of the foetus, as well as due to blood volume expansion and tissue growth of the mother [37]. Folate deficiency during pregnancy, especially around the time of conception, is strongly correlated with increased risk of neural tube defects such as spina bifida [38]. A recent study showed significant reductions in rates of both pre-eclampsia in mothers and SGA (small for gestational age) newborns with maternal folic acid supplementation (but no other associations between pregnancy and birth outcomes) [39].

*Vitamin D*: Vitamin D deficiency is estimated to affect one billion people globally and is increasingly recognized as being common amongst pregnant women [20]. Despite its important role in bone homeostasis, brain development and modulation of the immune system, the impact of antenatal vitamin D is still poorly understood [40], not least because of uncertainties with appropriate biomarkers and cut-off points. A systematic review suggested that women with circulating 25-hydroxyvitamin D (25(OH)D) concentrations <50 nmol/L in pregnancy have an increased risk of preeclampsia, gestational diabetes mellitus, preterm birth and SGA newborns [41]. A Cochrane review found a significant relationship between an increase in serum vitamin D concentrations at term and borderline reduction in low birthweight [42] but there is yet not enough evidence for policy as the number of high-quality trials is thought to be currently too small to draw conclusions on its usefulness and safety [18].

*Other vitamins*: Deficiencies of yet other vitamins such as vitamin B12 and perhaps vitamin A may be important but evidence is sparse or conflicting [22,43]. There was an earlier recommendation by the FAO (Food and Agricultural Organization)/WHO of a 40% increase in the vitamin B-12 dietary allowance to meet foetal demands and increased metabolic needs [21]. As pregnancy does not require additional vitamin E and it is common in most diets, additional vitamin E is unlikely to be required [21]. Observational or experimental data linking water-soluble vitamins to any risk of maternal mortality are apparently unavailable [21]; these vitamins, such as vitamin C, thiamin, niacin and riboflavin and others, do however appear to decline in serum or plasma levels, likely due to extra uptake by the foetus or haemodilution, or in the case of niacin also increased urinary excretion [21]. Most return to normal within a week of delivery [21]. Possible side-effects of overdosage of vitamin E and vitamin C in pregnancy are discussed below (Section 7.2).

## 4. Interactions among Micronutrients

Interactions between micronutrient-dependent physiological and biological actions can be both positive, e.g., zinc and vitamin A, and negative, as e.g., with zinc or copper and iron [43]. Addition of zinc to iron and folic acid supplements have been shown to attenuate or even negate the positive association with outcomes due to iron, probably related to the inhibitory role of zinc in iron absorption [44]. Maintaining a balance between antioxidants such as selenium, and pro-oxidants (such as iron can be) has also been described as desirable, beyond the need to meet recommended intakes [20].

Because pregnant women in resource-poor areas are at risk of multiple micronutrient deficiencies [43], both the effects of single micronutrient deficiencies, and multiple ones, as well as interactions among them, all need to be considered. As described above, micronutrient deficiencies during pregnancy are associated with adverse pregnancy outcomes, especially in women of lower socioeconomic status who tend to have more than one deficiency, and those of young age who are at risk of being undernourished and underweight [11,18]. When important clinically, such micronutrient/micronutrient interactions complicate public health recommendations and interventions, as some will be synergistic and some will be antagonistic [20,22]. Framing specific recommendations can be further complicated by human variability in uptake and utilization of micronutrients, and genomics and epigenetic changes due to early deficiencies during gestation. One recent meta-analysis e.g., strongly suggested the *MTHFD1* G1958A polymorphism appears to be associated with increased maternal risk for NTDs (neural tube defects) in Caucasian populations [45]. Neural tube defects present as a wide range of phenotypes and the aetiology is multifactorial “with a large number of unclear genetic components, environmental conditions, and their interactions playing critical roles” [45]. This seems likely possible also for other micronutrients important in pregnancy outcomes. Multi-micronutrient supplementation is discussed below.

## 5. Offspring of Micronutrient-Deficient Mothers

Although pregnancy is the focus of this review, the effects of micronutrient deficiencies on their offspring also need to be addressed as part of the mother-child dyad. Perhaps the most noteworthy natural experiment demonstrating this necessity came about as a result of the Dutch famine of 1944 which provided a unique opportunity to study the long term consequences of maternal nutritional status and health outcomes in offspring [46,47]. Before the famine ended in 1945, rations were as low as 500 Kcal per person [47]. Expectant mothers who were subjected to the famine became severely macro- and micronutrient deficient. The famine was directly observed to affect fertility, infant birth weight, maternal weight gain, and the development of the neonate’s central nervous system [47].

Assessing the impact of antenatal micronutrient status of pregnant women (especially when improved by supplementation) on the outcomes for their offspring is a challenge due to the need to follow the women through pregnancy and then the offspring, often in less than ideal settings for such research. In an important study from Nepal, intellectual functioning, including working memory, inhibitory control, and fine motor functioning among offspring at 7 to 9 years of age were positively associated with prenatal iron/folic acid supplementation in an area of high iron deficiency [8]. Related and similar findings of positive impact on the child of maternal antenatal supplementation have been found in Bangladesh [48], China [49] and in HIV-infected mothers in Tanzania [50]. A study from rural Viet Nam found that low maternal 25-hydroxyvitamin D levels in late pregnancy were associated with reduced language developmental outcomes at six months of age [40]. Maternal antenatal zinc supplementation may have beneficial long-term consequences for neural development associated with autonomic regulation of cardiovascular function in children at 54 months whose zinc-deficient pregnant mothers had received supplementation [51]. Even in areas of mild-to-moderate iodine deficiency, subtle reductions in the intelligent quotient of children in those areas may be reduced on average by 8–13.5 IQ points but can be corrected in populations by salt iodization [33]. On the other hand, there is increasing evidence of a positive impact of multiple micronutrient supplementation to deficient mothers on the growth and development of their offspring, although mechanisms are still unclear and findings inconsistent. This is probably because of different formulations and dosages of the supplements, rather than lack of effect.

## 6. Gestational Micronutrient Deficiencies and Later Risk of Chronic Disease

**Figure 1:**
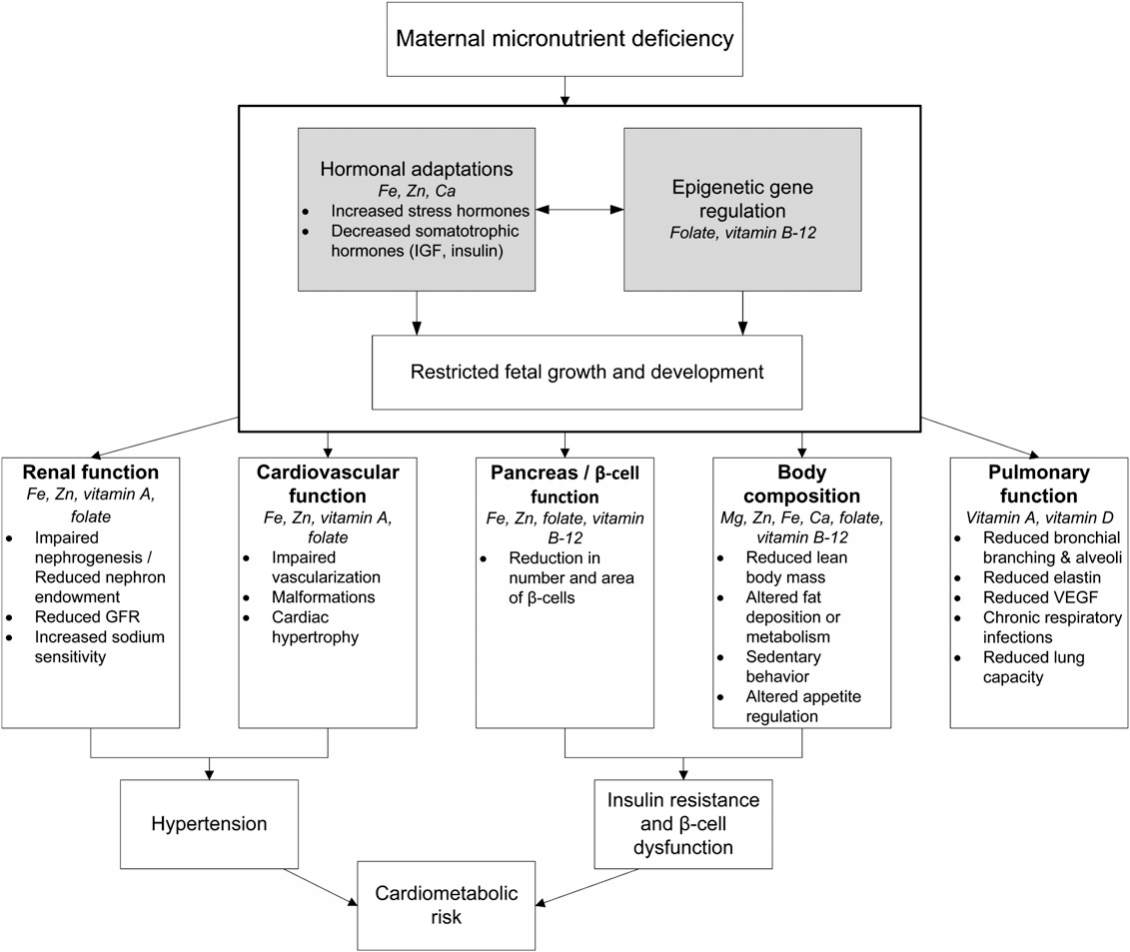
Conceptual framework by Christian and Stewart [52] for how maternal diet and micronutrient status may affect the development of chronic disease in the offspring. Gray boxes represent hypothesized pathways through which various micronutrient deficiencies may influence the growth, development, or function of the indicated systems.

It has been noted that whereas micronutrient deficiencies are known to be associated with various shorter-term adverse outcomes of pregnancy, their effects on long-term health and later chronic disease of the children of such pregnancies are largely unknown. It is now generally accepted that early life nutritional exposures, combined with changes in lifestyle in adult life, can result in an increased risk of chronic diseases [52,53]. The excellent review by Christian and Stewart [52] that considered various strands of evidence, including animal studies, concludes that there are also strong suggestive links between intrauterine micronutrient status and the potential risk of chronic diseases but the underlying mechanisms are largely unclear. However, it is known that micronutrient status in foetal and early life can alter metabolism, vasculature, and organ growth and function, and so is likely to have consequences for increased risk of cardiometabolic disorders, adiposity, altered kidney function, and ultimately type 2 diabetes and cardiovascular diseases [52]. Epigenetic influences, as mentioned previously, heritable long-term changes in gene expression which are not caused by changes in gene sequence [54], may also play a significant role in long-term pregnancy outcomes. Christian and Stewart [52] have suggested a conceptual framework for how maternal diet and micronutrient status may effect the development of chronic disease; the most likely, given present knowledge, seem to be vitamin A, folate, iron and zinc and perhaps calcium and magnesium as shown in their conceptual framework (Figure 1).

Numerous studies have found significantly higher morbidity and mortality rates amongst individuals who were exposed to the nutritional shortages during gestational developmental periods, compared to unexposed individuals as e.g., in the above-mentioned Dutch famine [46,55,56]. Exposure during early gestation was associated with increased glucose intolerance, increased prevalence of coronary heart disease, elevated atherogenic lipid profile, increased risk of obesity, increased risk of schizophrenia, and disturbances in blood coagulation [47,55,56]. Some of these morbidities were found to transcend generations, with higher rates of obesity and associated co-morbidities observed in second-generation offspring of males who were exposed to the famine prenatally [57,58]. It is known that nutritional deficiencies during pregnancy may result in epigenetic modifications to the human genome, resulting in changes to gene expression, without alterations to gene sequence. In the review by Christian and Stewart [52], of how deficient maternal micronutrient status might influence the development of chronic disease in the children, they suggest that micronutrient status might influence regulatory pathways, such as hormonal adaptations and epigenetic gene regulation that can influence restricted growth and development in the developing foetus (Figure 1). In population-based prospective cohort studies, maternal hyper-homocysteinaemia (a biomarker of folate deficiency) was, for example, also linked to a higher risk of adiposity and type 2 diabetes in mothers and their offspring, both of which have future negative outcomes, and in the shorter term, lead to negative birth outcomes [39].

An emerging trend, in terms of noncommunicable diseases, is that of increasing numbers of overweight and obese mothers such as is now happening in LMIC, often at the same time, as their underweight children—the so-called “double burden of malnutrition”. This is important because of increasing evidence e.g., from the Danish National Birth Cohort studies, that obese women have a higher risk of micronutrient deficiencies [59], and so micronutrient deficiencies associated with pregnancy in overweight/obese women are becoming increasingly an issue in both affluent and lower- and middle-income countries. High-dose supplementation with vitamin C and vitamin E has been found to not prevent preeclampsia but to increase the rate of babies born with low birthweight [59]. A later systematic review confirmed the lack of effect on preeclampsia and also found an increased risk of developing gestational hypertension and premature rupture of membranes, with a decreased risk of *abruptio placentae* [60,61]. There does not appear to be evidence to recommend supplementation with vitamins C and E.

## 7. Addressing the Problem

Short-term clinical interventions for micronutrient deficiencies may be critical for the immediate pregnancy and should obviously be addressed on presentation. Particularly in countries with under-resourced Health Systems, concurrent antenatal preventive measures also need to be in place and must be strengthened and scaled-up for optimizing maternal, neonatal and young child outcomes. The extended roles of other opportunities throughout the life course such as the adolescent young woman [28] are being increasingly recognized as further opportunities. The distinction will be made here between clinical interventions, those occurring during the pregnancy and delivery such as antenatal supplementation e.g., with iron and folic acid and obstetric care, and those occurring before the pregnancy and periconceptionally, such as preventive weekly iron and folic acid supplementation [62] which are seen as more public health and nutrition interventions.

### 7.1. Clinical

There have been significant strides in addressing perinatal nutritional issues, especially at the public health level. Consequently, the risk of nutritional insufficiency in pregnant women in most middle and high-income populations has been drastically reduced but remains a real risk in many LMIC women. Clinical care is used here in the sense of care before pregnancy, during the actual pregnancy, and during delivery and the neonatal phase. For convenience, “antenatal” refers to the pregnant women’s care, whereas “perinatal” is larger including the periconceptional time and even before, as well as including immediately after birth. In terms of micronutrients, these clinical interventions include oral tablet supplementation, improved diets and nutrition education, food supplementation, monitoring of anaemia levels where facilities permit, management of weight gain, and encouragement to plan for breastfeeding, and related activities such as delayed cord clamping to improve neonatal iron stores.

#### 7.1.1. Supplementation with Micronutrient Tablets

The Recommended Dietary Allowance RDA of iron increases by about 50% during pregnancy (from 18 mg to 27 mg). Due to the fact that the median dietary intake of iron in pregnant women is considerably lower than the Estimated Dietary Requirement (EAR) for iron, even in an affluent country like the USA, and often lower still in LMIC, it is recommended that pregnant women take iron supplements [63,64]. Antenatal iron and folic acid supplementation is a well-established intervention but the coverage and impact has been poor despite clear WHO recommendations for both anaemic and non-anaemic pregnant women and by national bodies [63,64,65]. Some other countries, especially more affluent ones, often have their own policies [64,65] including not recommending iron and folic acid at all in non-anaemic pregnant women. However, many women in such countries are anyway receiving a multiple micronutrient supplement [65], and there is a good body of research suggesting this should be recommended for pregnant women in LMICs [10,18,66] and there is growing support for the potential replacement of iron-folic acid supplements in pregnancy with multiple micronutrient supplements in populations at risk [18]. All bodies, multilateral or national, recommend supplementation to be complemented by healthy antenatal diets.

The reasons for the generally poor coverage of the existing recommended iron/folic acid tablets have been extensively reviewed [3]. They include: poor antenatal attendance especially in the first two trimesters; the effects of lower status of women; the taste and side effects of iron-containing tablets (especially lower quality ones); logistical issues; lack of conviction of positive impact by health workers; fear of larger babies where there is inadequate obstetric care; and so on [3]. Nevertheless, where coverage is good, the results are impressive and can be expected to have an impact on the mother’s health, reducing anaemia and improving birth outcomes, and a likely impact on infant and young child outcomes, as noted above. A study in rural western China found that antenatal supplementation with iron-folic acid was associated with longer gestation and a reduction in early neonatal mortality compared with only folic acid, whereas multiple micronutrient supplements were associated with a modest increased birth weight compared with folic acid [67]. Despite the weight gain, there was no significant reduction in early neonatal mortality [67]. In 2012, the World Health Assembly endorsed nutrition targets for 2025 that included a 50% reduction in the number of women of reproductive age affected by anaemia compared with 2011 [25].

There is accumulating evidence that folic acid by supplementation has an additional protective effect against adverse pregnancy outcomes, as well as the now well-established reduction in neural tube defects (NTD) [18]. Studies have suggested improved neurodevelopmental outcomes in children of mothers with higher blood folate concentrations or mothers receiving antenatal folic acid supplements [37]. The RDA increases by 50% during pregnancy, from 400 μg/day to 600 μg/day [20]. This is hard to accomplish without consumption of folic acid-fortified foods or folic acid supplements. For this reason, periconceptional supplementation of 400 μg/day of folic acid is recommended internationally for women of childbearing age in order to minimize neural tube defects [20]. Except in some settings, coverage has not usually been impressive, especially to those of poor socio-economic status and adolescents who are amongst those most at risk of unplanned pregnancies. For example, the CDC reports that about 50% of U.S. pregnancies are unintended and most women are not aware that they are pregnant until they are about two months into the pregnancy. However, the neural tube closes between 23–27 days after conception [20], and so this is already too late. An alternative approach that has been considered is to recommend folic acid supplements for all women who are of child-bearing age. However, long term folic acid supplementation on the off chance that an unplanned conception occurs has significant implementation and cost implications. Where pregnancies are generally carefully planned, as was the case in one experience in China, folic acid supplementation has been successful. However, the public health food-based population approach of fortification is now the accepted intervention in most countries, and where instituted, has shown a dramatic reduction in the incidence of NTDs [38].

There are also several other clinical considerations to be made when supplementing with folic acid. For example, folate may mask symptoms of megaloblastic anemia, which could be an indicator of vitamin B-12 deficiency [68]. Indicators of low vitamin B-12 are associated with adverse pregnancy outcomes, anaemia, low birthweight, and intrauterine retardation [9]. By the possible masking of vitamin B-12 deficiency, folic acid could make it difficult to detect and remedy B-12 deficiency, but the evidence for this happening with fortification of cereal staples is mixed. Oral supplementation of urban Indian women with vitamin B-12 throughout pregnancy and early lactation significantly increased vitamin B-12 status of both mothers and infants [9] and so worth considering where vitamin B12 intakes are marginal. There are also concerns from observational data of possible increases in rates of some cancers with higher levels of folic acid in the diet due to the relatively high levels used in fortification [20].

As noted, WHO now recommends calcium supplementation to reduce the risk of hypertensive diseases in pregnancy. However calcium requires large and frequent tablets, as well as the 60 mg of iron and 400 µg of folic acid and where the iron and calcium may negatively react intra-intestinally [34]. Recently a novel micronutrient powder containing micro-encapsulated pH-sensitive calcium in addition to iron and folic acid has been designed to facilitate early intestinal release of the iron and delayed calcium release [34].

In affluent countries, where supplementation with antenatal multimicronutrient supplements is relatively common and often recommended [65], use of targeted antenatal micronutrients probably has the potential to decrease infant morbidity and mortality in anaemic and deficient women, especially low-income urban women [69]. In a Canadian study, self-reported vitamin supplementation was associated with decreased odds of miscarriage although other associated positive health-related behaviours also likely contributed [70]. On the other hand, an earlier systematic review found that taking vitamin supplements prior to pregnancy or in early pregnancy, did not prevent miscarriage or stillbirth but the mothers appeared to be less likely to develop pre-eclampsia (but were more likely to have a multiple pregnancy) [71]. The evidence is not yet entirely clear, as iron and folic acid supplements in Indonesian women significantly reduced the risk of early neonatal death [72]. Similarly a double-blind cluster-randomized trial, also in Indonesia, found that maternal multiple micronutrient supplementation, as compared with iron/folic acid, reduced early infant mortality, especially in undernourished and anaemic women [73]. The size of the pregnant women appears to modify the effect and well-nourished women may be less appropriate for supplementation where there is inadequate clinical support.

Supplementation with multiple micronutrient formulations has a certain logic to it given the multiple micronutrient deficiencies that frequently occur together and the interactions between micronutrient-dependent physiological and biological actions [43]. Current evidence has been described as suggesting that vitamins and minerals have added bio-functionality which may be particularly important in pregnancy with synergisms e.g., between folic acid and vitamin B12, possibly enhancing their biological potential—in this case to further reduce the occurrence of NTDs [20].

An independent systematic review and meta-analysis of 12 randomized, controlled trials comparing multiple micronutrient supplementation with iron-folic acid supplementation found that both supplements were equally effective in reducing anaemia (even though iron content was often lower in the multimicronutrient supplement) and resulted in a small, significant increase in mean birthweight; larger micronutrient doses appeared to have a greater impact [10]. The findings of other intervention trials, especially effectiveness trials, have been variable, although efficacy is largely accepted. Many factors affect the impact, such as baseline iron and/or anaemia levels, diet, the disease environment and importantly it seems the size of the pregnant woman. A recent study in Chinese women e.g., showed that compared with controls taking folic acid, prenatal iron/folic acid or multiple micronutrient supplements improved iron status later in pregnancy but did not affect perinatal anaemia in women with no or mild anaemia [74].

Nevertheless, “despite encouraging high compliance to community-based supplementation, a proportion of mothers remain anaemic, suggesting a need to also address parasitic and other infections and malaria” [10,13]. It is also worth noting that meaningful improvements with antenatal multiple micronutrients in height and cognitive development in children by two years of age have been observed, although these findings have been less consistent. Nevertheless, following these findings, it was concluded that replacing iron-folic acid supplements with multiple micronutrients in the package of health care, including improved obstetric care of health and nutrition interventions, would improve the impact of supplementation on birthweight, small-for-gestational age neonates, and perhaps child growth and development [10]. Despite some initial concern in some settings of (non-significant) risk of increased neonatal mortality [75] (not found in other reviews e.g., Haider *et al.* [66]), the conclusion immediately above was later endorsed by the Lancet Series (2013) following further evidence supporting the approach [18]. Trials are underway in a number of countries at present. Supplementation with multivitamins (vitamin B complex, vitamin C and vitamin E) also significantly decreases the risk of adverse pregnancy outcomes among HIV-infected women [76].

A consensus appears to be emerging on the usefulness of antenatal supplementation with multimicronutrients, especially for improvements in birthweights [10,18], at least in LMIC. Replacement of iron-folic acid with multiple micronutrient supplements in pregnancy, as a public health recommendation in at-risk populations, seems warranted, although further evidence from effectiveness assessments might be needed to guide a universal policy change [18]. However, delivery platforms for micronutrient antenatal care are a constraint in many settings e.g., a recent study in PNG showed how socio-cultural, health care staff attitudes and economic factors all affect antenatal care attendance and that only a third of women receive any antenatal care during pregnancy [77]. Clearly, however effective micronutrient interventions are for pregnant women, other factors, including logistics need to be addressed in many settings.

#### 7.1.2. Food Supplementation

Food supplementation, especially in emergency and resource-poor settings is increasingly evidence-based. Emergency rations and supplies in particular have invested considerable resources in ensuring that the micronutrient content of such supplements are adequate while recognizing that in undernourished pregnant mothers it is the low energy (caloric) content of the available diets that is the main risk. The MINIMat randomized trial in Bangladesh tested the hypothesis that antenatal multiple micronutrient supplementation and an early invitation to food supplementation would improve birth outcomes [78]. They found that among these pregnant women from poor communities, supplementation with multiple micronutrients, as well as just iron and folic acid, combined with food supplementation, resulted in decreased childhood mortality. A recent review concluded that a dietary pattern containing several protein-rich food sources, fruit, and some whole grains is associated with a reduced risk of preterm delivery [1]. A platform used with limited experience (in pregnant women) has been the use of multimicronutrient powders (added to food) during the antenatal period, or more recently lipid-based supplements that supply both dietary energy, protein and micronutrients [79]. Studies show that use of micronutrient fortified supplementary foods, especially those containing milk and/or essential fatty acids during pregnancy, increase mean birthweight by around 60–73 g [80]. Fortified food supplements containing milk and essential fatty acids, along with micronutrients, offer benefits for improving maternal status and pregnancy outcome [80]. Fortified beverages containing only multiple micronutrients have been shown to reduce micronutrient deficiencies such as anaemia and iron deficiency. Food supplementation, while clearly effective in undernourished mothers will not be discussed further here, as it is mainly an intervention to increase dietary energy and the micronutrients needed to accompany it are largely known. Other antenatal *clinical* advice and monitoring, while clearly essential to the mother’s health and reproductive outcomes, are also not discussed here but recognized as part of the larger care of the mother, of which adequate micronutrient status is but one part.

### 7.2. Public Health Measures

Public health and nutrition measures aim to improve pregnancy outcomes in general, especially for those women with limited access to good antenatal clinical care, and to reduce the risk of periconceptional micronutrient deficiencies, among other ancillary benefits. The most commonly used approaches, in terms of micronutrients includes: blanket supplementation, and sometimes targeted supplementation, policies and programmes; fortification; and, general measures to improve diets and micronutrient intakes and general health.

#### 7.2.1. Supplementation

Public health supplementation includes blanket approaches e.g., all women of reproductive age, especially adolescents, receiving weekly iron and folic acid supplements [62]. The use of weekly iron and folic acid supplements through schools or factories has proven efficacy and is recommended by the WHO [62]. As a preventive measure it has not been taken up by governments and requires more implementation experience in national programmes, despite already promising experiences in some countries such as the Philippines and Vietnam. Targeted, preconceptional supplementation such as folic acid supplements for young women intending to get pregnant has had limited use and success, not least because the majority of pregnancies are not planned, as discussed above. Targeted iron/folic acid supplementation to pregnant women has a long history and continues to be recommended nationally and by the WHO but has been relatively unsuccessful due to poor covergae, especially in LMIC, as also discussed above.

Oral iodized oil has also been used for this purpose but as a public health measure has been supplanted by iodized salt programmes. Where coverage by iodized salt is sub-optimal, WHO recommends that pregnant (and lactating) women should be given an oral supplement of iodized oil [81]. Although 38 million newborns are born iodine-deficient in LMIC, affluent countries are also increasingly at risk unless supplementary measures e.g., iodine fortification of bread or supplementation of pregnant women are undertaken. Even in affluent countries such as the UK and Australia, poor iodine intake in pregnancy predicts lower child IQ [82] and in a small recent study in South Eastern Australia, less than half (46%) of pregnant women were following national recommendations and only 18.5% believed they needed a supplement and only a third (34.5%) had been given adequate advice by their medical practitioner [83]. In the USA, iodine supplements are used by only 22% of pregnant women [82]. Such findings explain the continued global emphasis on salt fortification with iodine [84], despite the challenges of other recommendations for populations to reduce their salt intake to reduce the prevalence of hypertension.

#### 7.2.2. Nutrition Education, Dietary Improvement and Improved Public Health Measures

Other interventions that impact on the micronutrient status during pregnancy include dietary measures and other public health and social interventions such as deworming, education and horticultural activities. While the risk of being born low birthweight is significantly greater with moderate preconception anaemia [85], it has also been noted that in many unsafe settings, mothers purposefully “eat-down” aiming to have a smaller neonate. A failure of nutrition education has also been implicated in poor diets as well as some dietary taboos [21] and soil-transmitted helminthes [86]. Nevertheless, where access and availability to foods is possible, improving diets by including such items as eggs and animal-source foods are likely to provide protein, energy and micronutrients. However, such foods are not often available to the very poor, or there are cultural constraints, which is why food supplements to such pregnant women is now a recommendation [3,18].

International guidelines recommend routine safe and protective prevention and treatments, during pregnancy, to reduce hookworm, malaria and other infections such as schistosomiasis [87]. Despite the effectiveness of such programmes, and because women with high levels of hookworm or malaria infections are at high risk of anaemia [86], there continues to be a need for more general scaling-up of coverage in affected populations. A recent randomized trial (that included pregnant women with anaemia and iron deficiency at baseline) in a malaria endemic area found major gains in birthweight, without apparent effect on *Plasmodium* infection and urged that universal coverage of iron supplementation (60 mg per day) should be scaled-up, preferably with cover by IPT (intermittent preventive treatment of malaria) [88]. A systematic review and meta-analysis recently concluded that more evidence is needed to the long-standing, and often contentious, role of giving iron in malaria-endemic populations and so concluded that currently it is prudent to provide iron in combination with malaria prevention during pregnancy [89]. There is increasing consensus in this view, along with the need for concomitant improved obstetric care and diet.

Attention to adolescent girls as an important preventive strategy is increasingly recognized, despite some strong cultural and social constraints. It has been observed that, even in affluent settings, adolescents are more likely than adults to consume energy-dense, micronutrient-poor diets and to have adverse pregnancy outcome such as increased risk of SGA [16]. The risk is likely to be even greater in food-insecure populations such as in Central Africa [90]. Other non-direct micronutrient interventions that could be expected to have a positive impact on nutrition and health of pregnant women (at least where most births are within a marital relationship), include interventions to increase the age at marriage and first pregnancy are important, and can reduce repeat adolescent pregnancies by 37% [18]. The African Union has recently launched a new campaign to end child marriage in Africa [91] which, if successful would be expected to have positive reproductive outcomes.

Table 1, derived from Bhutta *et al.* [18], shows micronutrient interventions that have an adequate evidence base to be recommended for women of reproductive age and during pregnancy (as well as maternal supplementation with balanced energy and protein including through supplementation).

#### 7.2.3. Fortification

Fortification can be considered a dietary intervention and one that has recently been recommended by the Lancet Maternal and Young Child Undernutrition series and has been in practice for over sixty years in many affluent countries [18]. Bhutta *et al.* [18] concluded that “fortification has the greatest potential to improve the nutritional status of a population when implemented within a comprehensive nutrition strategy, including for pregnant women and has the advantage of reaching women before pregnancy”. Iodized salt programs are now implemented in many countries worldwide, and the past two decades have shown considerable progress, so that globally, 76% of households are now adequately consuming iodized salt. On the other hand, nearly 30% of school-aged children are estimated to have insufficient iodine intakes and global progress appears to be slowing [84]. The need for continual global scaling-up and consolidation of existing programmes has already been commented upon. There have also been efficacy, and limited effectiveness studies of doubly fortified salt with iodine and encapsulated iron.

The provision of balanced energy protein supplementary foods to underweight pregnant women was also considered to have enough evidence of reduction in SGA and stillbirths and improved birthweights for widespread implementation, whereas maternal vitamin D and zinc supplementation, while promising, were considered to have insufficient evidence [18].

Fortification of cereal flours with iron and often other micronutrients such as some B group vitamins, and more recently zinc and even selenium, has been in existence e.g., in the USA, for over 60 years, and now 80 countries globally have legislation to mandate fortification of at least one industrially milled cereal grain (79 countries have legislation to fortify wheat flour; 12 countries to fortify maize products; and five countries to fortify rice) [38]. Costa Rica is the only country that mandates fortification of all three grains, and Papua New Guinea is the only country that requires only rice fortification. Currently the 79 countries that mandate required fortification of wheat flour produced in industrial mills require at least iron and folic acid, except Australia, which does not include iron, and Congo, the Philippines, Venezuela, and the United Kingdom, which do not include folic acid. Additionally, seven countries fortify at least half their industrially milled wheat flour through voluntary efforts and it has been estimated that about a third (31%) of the world’s industrially milled wheat flour is now fortified with at least iron or folic acid through these mandatory and voluntary efforts [38]. Other success stories include the fortification of sugar with vitamin A in Central America. A continuing challenge is that populations most at risk of deficiency either cannot afford fortified foods or, especially in lower-income countries, they are not available to them. Nevertheless, fortification is likely to be an increasingly major part of the reduction of micronutrient deficiencies, including during pregnancy.

**Table 1:** Nutrition/micronutrient interventions for women of reproductive age and during pregnancy (based on Bhutta *et al.* in the Lancet series of 2013 which has estimates of size of the significant effects and the evidence from which they come [18]).

Intervention	Setting	Comments (Only Significant Findings and Original Systematic Review References)
***Folic acid supplementation***		
WRA *	LMIC ** and affluent countries	[92]
Pregnant women	Mostly more developled countries	[93]
***Iron and iron-folic acid supplementation***		
WRA	Both LMIC and affluent countries. Interventions mainly given in school settings to adolescents and evidence mostly from effectiveness studies	Intermittent iron supplementation (once or twice a week)—reduces anaemia rates [94]
Pregnant women	Both LMIC and affluent countries. Intervention	Reduction in LBW ***, reduction in anaemia rates at term and improved Hb [95]
***Multiple micronutrients (MMN) supplementation***		
Pregnant women	LMIC and affluent countries. Studies compared MMN with two or fewer micronutrients	Reduction in LBW and currently insignificant data for neurodevelopmental outcomes in offspring [96]
***Calcium supplementation***		
Pregnant women	LMIC and affluent countries. Mostly effectiveness trials	Reduction in pre-eclampsia as well as LBW and pre-term birth [97]
***Iodine through salt iodization programmes***		
Pregnant women	Mostly LMIC. Mostly effectiveness trials.	Cretinism at 4y reduced, improved birthweight, developmental scores higher in young children [98]

* WRA = women of reproductive age; ** LMIC = Low- and Middle-Income Countries; *** LBW = Low birth weight.

## 8. Conclusions

The deficiencies in micronutrients that affect many women of reproductive age are now known to be associated with adverse maternal and perinatal outcomes. These adverse outcomes can have longer-term impacts into adulthood [18,99,100]. Personal, social and economic costs are high [3]. Maternal undernutrition has been described as one of the most neglected aspects of nutrition in public health globally [99,100]. Consequently, low-cost public health interventions that might help to ameliorate the impact of poor nutrition and diets, high disease burdens and the socio-cultural factors contributing to the high levels of these micronutrient deficiency problems during pregnancy, and before, continue to need scaling-up in scope and coverage [101].

Important factors besides inadequate diet and diseases that are indirectly related to maternal, foetal, and neonatal nutritional status and pregnancy outcomes include young age at first pregnancy and repeated pregnancies. Young girls who are not physically mature enter pregnancy with depleted nutrition reserves and often anaemia [102] and other micronutrient deficiencies [3,43]. While micronutrient deficiencies can undoubtedly have profound influences on the health of the mother and her child, there remain considerable areas of uncertainty and controversy that has made the development of robust public health recommendations a challenge [20]. Along with the noted challenges to get compliance, especially periconceptionally and in settings with limited health care capacity, and questions of how optimal micronutrient formulations and dosages are established, Berti *et al.* [20] have called for “adequately powered, randomized controlled trials with long periods of follow-up” to “establish causality and the best formulation, dose, duration and period of supplementation during pregnancy”. However, the methodological issues in doing this would be considerable, especially in establishing causality. Consequently, factors that are known to be important, such as entering a pregnancy adequately nourished, being aged beyond adolescence, and good health and obstetric care, and nutrition education and support, should be scaled-up actively in the meantime.

If proven to be effective and safe in national health care systems, supplementation with multimicronutrients, at least in pregnancy, could complement preventive supplementation with weekly iron and folic acid in vulnerable populations. This could help break the intergenerational reality of low birthweight infants growing up disadvantaged and stunted and so at high-risk of repeating the same cycle. Whereas there has been a lot, if insufficient, attention paid to iron deficiency anaemia in pregnant women, most of the other involved micronutrients, are less well characterized [20,43]. Micronutrients likely to be important for maternal, infant and child outcomes include iron, iodine, folate, vitamin B12, vitamin D, calcium, and selenium, probably zinc and maybe others, along with appropriate dietary energy intakes. In addition to programmes to reduce micronutrient deficiencies such as micronutrient supplementation and food fortification, needed complementary interventions should optimally improve overall maternal nutrition, address household food insecurity, reduce the burden of maternal infections such as HIV and malaria, improve sanitation, and actively address gender and social disadvantage.
